# Increased Incidence of Glaucoma in Sensorineural Hearing Loss: A Population-Based Cohort Study

**DOI:** 10.3390/ijerph16162907

**Published:** 2019-08-14

**Authors:** Hsiang-Wen Chien, Pei-Hsuan Wu, Kai Wang, Chi-Chin Sun, Jing-Yang Huang, Shun-Fa Yang, Hung-Chi Chen, Chia-Yi Lee

**Affiliations:** 1 Institute of Medicine, Chung Shan Medical University, Taichung 402, Taiwan; 2 Departments of Ophthalmology, Sijhih Cathay General Hospital, New Taipei City 221, Taiwan; 3 Department of Ophthalmology, Cathay General Hospital, Taipei 106, Taiwan; 4 Department of Otolaryngology–Head and Neck Surgery, Tri-Service General Hospital, Taipei 114, Taiwan; 5 Department of Ophthalmology, Chang Gung Memorial Hospital, Keelung 204, Taiwan; 6 Department of Chinese Medicine, Chang Gung University, Taoyuan City 333, Taiwan; 7 Department of Medical Research, Chung Shan Medical University Hospital, Taichung 402, Taiwan; 8 Department of Ophthalmology, Chang Gung Memorial Hospital, Linkou 333, Taiwan; 9 Department of Medicine, Chang Gung University College of Medicine, Taoyuan 333, Taiwan; 10 Center for Tissue Engineering, Chang Gung Memorial Hospital, Linkou 333, Taiwan; 11 Department of Ophthalmology, Show Chwan Memorial Hospital, Changhua 500, Taiwan; 12 Department of Optometry, College of Medicine and Life Science, Chung Hwa University of Medical Technology, Tainan 717, Taiwan

**Keywords:** glaucoma, sensorineural hearing loss, epidemiology, degeneration, neuropathy

## Abstract

The purpose of the current study was to evaluate the incidence of glaucoma in patients diagnosed with sensorineural hearing loss (SNHL) via the application of the National Health Insurance Research Database in Taiwan. A retrospective cohort study was conducted. Patients with a diagnosis of SNHL were enrolled in the study group after an exclusion procedure and a propensity score matched group without SNHL was served as the control group with a 1:2 ratio. The main outcome was regarded as the emergence of glaucoma diagnostic codes. Cox proportional hazard regression was applied to analyze the incidence and adjusted hazard ratio (aHR) of glaucoma in the multivariate model. A total of 15,686 patients diagnosed with SNHL were enrolled in the study group while another 31,372 non-SNHL individuals served as the control group. There were 444 glaucoma events in the study group and 647 glaucoma events in those non-SNHL individuals after the follow-up interval of 16 years. The study group demonstrated a significantly higher aHR compared to the control group after adjusting for multiple possible risk factors. In the subgroup analysis, both the normal tension glaucoma and angle closure glaucoma subgroups revealed a higher aHR in the study group. In conclusion, the patients with SNHL demonstrated a higher incidence of developing glaucoma. Moreover, the incidence was more prominent for patients diagnosed with normal tension glaucoma and angle closure glaucoma.

## 1. Introduction

Sensorineural hearing loss (SNHL) refers to hearing impairment resulting from damage among cochlear, labyrinth and central nervous system [1,2]. SNHL influences the majority of the population for which the prevalence of age-related SNHL accounted for more than 60 percent in those older than 65 years [3]. Regarding the etiology of SNHL, various causes including noise-related, infectious, autoimmune ear disease, drug toxicity and vascular lesions can lead to SNHL [2,4], and SNHL can also be a presentation of vestibular schwannoma, which must be excluded by magnetic resonance examination [5]. In addition, central nervous system lesions like multiple sclerosis can cause the development of SNHL [6].

Certain neurodegenerative disorders have been proven to occur with SNHL concurrently [7]. Alzheimer’s disease, a dementia characterized with memory impairment, executive dysfunction and the combined presence of amyloid and tau [8], showed a higher risk of SNHL occurrence in a previous study [9]. In addition, SNHL has been found to be associated with other brain degeneration such as cognitive impairment and general dementia in different studies [10]. As the above neurological defects share the same clinical manifestation of neurodegenerative process with SNHL [2,8,10], a general neurological defect of other organ in such populations may exist.

Glaucoma is the progressive optic neuropathy that presents with visual field defect and elevated intraocular pressure (IOP) in the majority of patients which is the most common reason of irreversible blindness throughout the world [11]. Although glaucoma can be further divided into three subtypes, namely, open angle glaucoma (OAG), normal tension glaucoma (NTG) and angle closure glaucoma (ACG) [12], the degeneration of the optic and retinal nerve tissue is the common manifestation among glaucoma individuals [13,14]. However, there is barely any evidence that indicates the relationship between SNHL and glaucoma. Since both diseases share similar features about neurological degeneration, we wonder whether there is a higher chance that the two disorders occur in the same population.

Herein, we aimed to investigate the incidence of glaucoma in individuals diagnosed with SNHL via the application of the National Health Insurance Research Database (NHIRD) in Taiwan. In addition, the incidences of different glaucoma subtypes between individuals with and without SNHL were also compared in the multivariate analysis.

## 2. Materials and Methods 

### 2.1. Data Source

The retrospective population-based cohort study was conducted in accordance with the Declaration of Helsinki, and the protocol was approved by both the National Health Insurance Administration and the Ethics Committee of Chung Shan Medical University (Project identification code: CS-17075). In addition, the need of informed consent was waived by the Chung Shan Medical University. Supported by the Taiwan National Health Research Institutes, the NHIRD contains medical information of insurance claims from nearly all the population in Taiwan. Those claims data were obtained from the Longitudinal Health Insurance Database 2005 version (LHID 2005) in the current study. The LHID 2005 includes information on two million individuals which were randomly selected from the NHIRD documents in the year 2005. The LHID 2005 were collected from 1 January 2000 to 31 December 2016, and the International Classification of Diseases, Ninth Revision (ICD-9), as well as the International Classification of Diseases, Tenth Revision (ICD-10) were applied for disease identification and diagnosis. Information regarding the medications prescribed to the patients, demographic data, socioeconomic condition, and residence of the patients is also accessible from the NHIRD. 

### 2.2. Patient Selection

Subjects were regarded as suffering from SNHL if their medical documents demonstrated (1) a diagnosis of SNHL, (2) the arrangement of pure-tone audiogram before the diagnosis of SNHL, and (3) the SNHL was diagnosed by the otolaryngologist. The index date was defined as the day of SNHL diagnosis. To evaluate the correlation between SNHL and glaucoma more precisely, certain exclusion criteria were used to exclude several impaired ocular status: (1) Diagnosed with legal blindness at any time; (2) diagnosed with ocular tumors before the index date; (3) arrangement of eyeball removal surgery or diagnosed with anophthalmos before the index date; (4) diagnosed with deafness at any time; (5) diagnosed with otological tumors before the index date; (6) arrangement of labyrinth removal surgery before the index date; (7) diagnosed with glaucoma and glaucoma suspect, optic neuropathy and age-related macular degeneration before the index date; (8) the diagnosis of SNHL was earlier than 2005, and (9) age younger than 20 or older than 100. Besides, every subject in the study group was propensity score-matched with two individuals without SNHL, which comprised the control group. Patients with SNHL that could not be matched with non-SNHL individuals were eliminated from the current study. 

### 2.3. Main Outcome Measurement

The presence of glaucoma was defined as the primary outcome according to (1) the emergence of glaucoma-related ICD-9/ICD-10 diagnostic codes after the index date, (2) the receipt of an optical coherence tomography exam before glaucoma diagnosis, and (3) the receipt of a visual field test before glaucoma diagnosis. Those glaucoma-related diagnostic codes that indicate clear underlying etiology (e.g., glaucoma due to ocular trauma and drug-induced glaucoma) or glaucoma suspect (e.g., ocular hypertension and anatomical narrow angle) were eliminated from the current study to prevent confusion as well as overestimation. Besides, only subjects that received those glaucoma diagnostic codes by an ophthalmologist were recognized as having achieved the primary outcome and were included in the study.

### 2.4. Demographic Data and Co-Morbidities

To ensure the health status of each participant was as homogenous as possible, we also evaluated the influences of age, gender and the following systemic co-morbidities in the multivariate analysis model: Hypertension, diabetes mellitus, ischemic heart diseases, hyperlipidemia, congestive heart failure, cerebrovascular disease, dementia, Alzheimer's disease, Parkinson's disease, liver disease, rheumatic disease, kidney disease, and hemiplegia or paraplegia. We traced the data in the database longitudinally from the index date of each subject to (1) the date of glaucoma diagnosis, (2) withdrawal from the National Health Insurance program, or (3) the date 31 December 2016.

### 2.5. Statistical Analysis

SAS version 9.4 (SAS Institute Inc, Cary, NC, USA) was used for the analyses mentioned in the current study. After propensity-matching with 1:2 ratios of both the study group and control group, a Poisson regression was conducted to calculate the incidence rate and the 95% confidence intervals (CI). Then, we used multiple Cox proportional hazard regression to yield the adjusted hazard ratios (aHR) by incorporating all the demographic information as well as systemic co-morbidities. The aHR of each demographic group and systemic co-morbidity were also analyzed. In the next step, the whole glaucoma population was divided into three subgroups: the OAG group, the NTG group and the ACG group. Following this, the influence of SNHL on the development of each glaucoma subgroup/subtype was analyzed separately. The subgroup analysis according to the age, gender and duration of SNHL-based subgroups in the study group was conducted. We drew Kaplan–Meier curves to demonstrate the cumulative incidence probability of glaucoma between the study and control groups, then applied the log rank test to evaluate whether a significant difference existed between the two survival curves. As almost all individuals in the NHIRD are Han/Chinese population, ethnicity was not considered as a confounding factor in the current study. Statistical significance was regarded as *p* < 0.05.

## 3. Results

**Figure 1:**
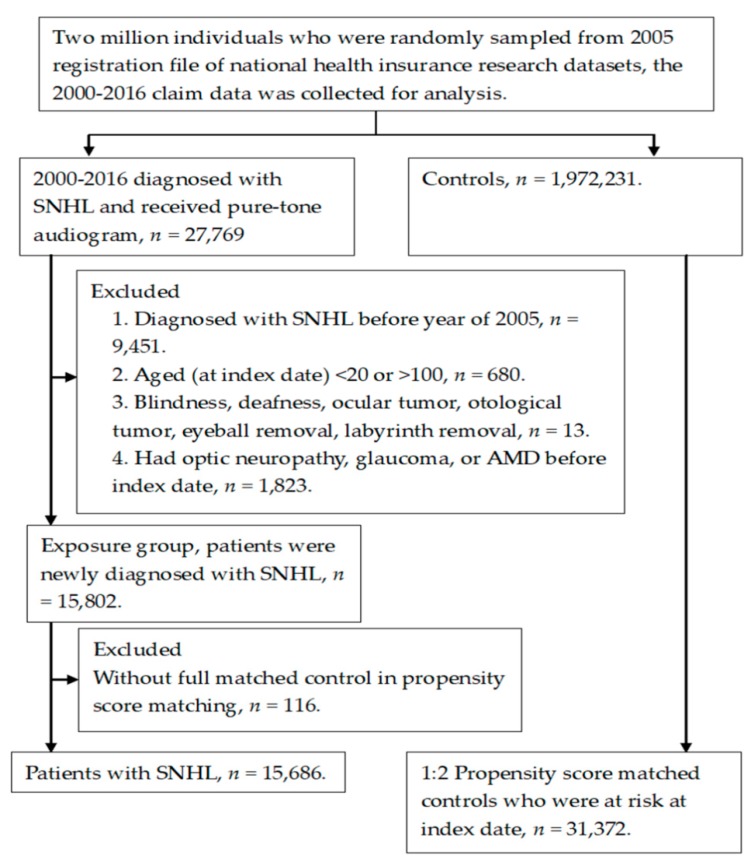
Flowchart of patient enrollment with and without dermatologic vasculature disease.

A total number of 15,686 patients diagnosed with SNHL were included in the study group, and another 31,372 non-SNHL individuals served as the control group. The flow chart of subject selection is shown in Figure 1. The numbers of people diagnosed with Parkinson's disease was significantly higher in the study group, while the individuals diagnosed with hypertension was prominently higher in the control group, and the remaining basic characters, including age and gender distributions, remained similar (Table 1).

**Figure 2:**
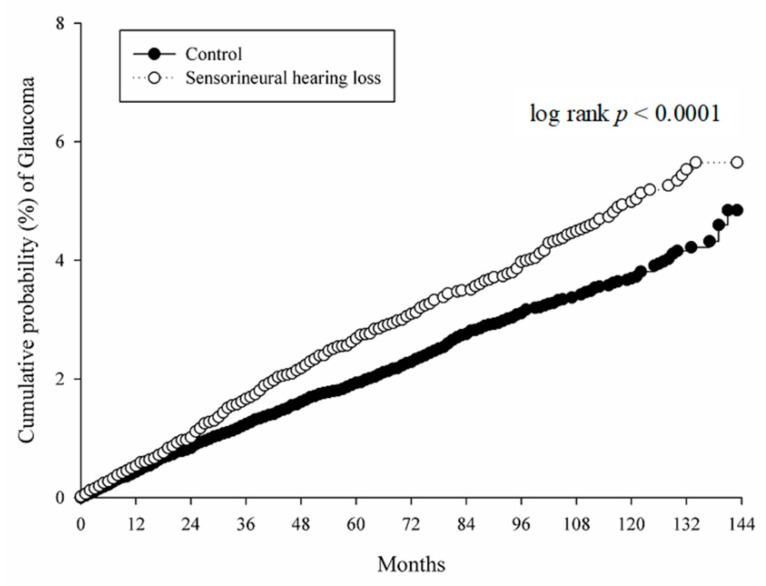
Kaplan-Meier curves with cumulative proportion of subconjunctival hemorrhage in the propensity score matched study and control groups.

After a follow-up period of up to 16 years, 444 glaucoma events occurred in the study group and 647 glaucoma episodes emerged in those non-SNHL individuals with a prominent crude relative risk in the study group (Table 2). In addition, the study group demonstrated a significantly higher aHR compared to the control group after adjusting for multiple possible risk factors (aHR: 1.318, 95% CI: 1.168–1.487). Other prominent risk factors for glaucoma development included being aged 60–79 years old, having ischemic heart disease and hyperlipidemia (Table 3). A higher cumulative probability of glaucoma was also found in the study group by the Kaplan-Meier curve (log-rank *p* < 0.0001, Figure 2).

In the glaucoma subgroup analysis, both the NTG and ACG subgroups revealed a significantly higher aHR in the study group, while the OAG subgroup only showed a marginally higher incidence (Table 4). The other subgroup analysis stratified by age, gender and duration of SNHL did not find any trend of glaucoma development among different subgroups (Table 5).

## 4. Discussion

Briefly, the current study demonstrated a significantly higher chance of glaucoma development in individuals diagnosed with SNHL after multivariable analysis. In addition, the NTG and ACG subgroups showed a significantly higher aHR among the general glaucoma disorders. Other potential risk factors for the occurrence of glaucoma include age and certain cardiovascular diseases.

Concerning the pathophysiology of SNHL, multiple factors are correlated to the neurological hearing impairment [2,15]. According to previous reports, advanced age is a risk factor for SNHL which is also known as age-related hearing loss or presbycusis [1,16]. In addition, vascular lesions such as transient ischemic attacks would cause SNHL, particularly the sudden SNHL subtype [17,18]. Furthermore, the elderly is vulnerable to the glaucoma-induced damage, which has been well-established in previous studies [19,20]. Moreover, the etiologies of glaucoma also include vascular impairment [21], which is a more prominent factor in NTG [22]. Regarding histopathological presentation, impaired hairy cell, spiral ganglion cells loss and cochlear-nerve/hair-cell synapses degenerations were found in those with SNHL but without a regular pattern [23]. In more detail, different degrees of cells loss were observed in SNHL with different etiologies: The noise and ototoxic drugs-related SNHL mainly result from both outer and inner hair cells damage [24], sudden SNHL featured with progressive loss of spiral ganglion neurons [25], and age-related SNHL presented with degeneration of both sensory and ganglion cells [26,27]. It seems that the degradation of spiral ganglion neurons may be more likely to be associated with glaucoma development because diminishment of ganglion neurons in retina had been observed in patients with glaucoma, while the sensory cells (i.e., photoreceptor) in such patients remain intact [28]. On the other hand, SNHL is associated with other neurodegenerative disease including Alzheimer’s disease and cognitive defects [3], in which degenerated nervous system were observed in both SNHL and several central nervous system impairments [8,23]. Similarly, the patients with glaucoma also showed a higher incidence of dementia, including Alzheimer’s disease [29,30]. Moreover, Ménière's disease, characterized by low- -to-medium frequency SNHL [31], shares analogous etiologies with glaucoma, including ganglion cell damage and pressure accumulation [32,33]. Since both diseases share similar risk factors, neurodegenerative features and co-morbidities, it is possible that patients with SNHL own a vulnerable nervous system in which the optic nerve could also be injured more easily than members of the normal population, leading to glaucoma development, as demonstrated in the current study.

Concerning SNHL and ocular diseases, age-related SNHL has been reported with a higher occurrence rate in those with age-related macular degeneration [34], while optic neuropathy and SNHL presented with same gene defect [35]. However, few studies have focused on the correlation between glaucoma and SNHL. In the current study, patients with SNHL demonstrated a significantly higher incidence of glaucoma development than those non-SNHL counterparts with a higher aHR and cumulative probability. To our knowledge, this is the first time the higher incidence of glaucoma in those diagnosed with SNHL compared to non-SNHL individuals has been illustrated. In addition to the enrollment of multiple potential risk factors of glaucoma into the multivariate model, we excluded those diagnosed with preceding glaucoma and optic nerve/retina-related degenerative diseases to prevent overestimation of glaucoma development and the misleading of time sequence. Moreover, the higher cumulative probability in the study group revealed that the glaucoma occurred in patients with SNHL annually, rather than an acute stress episode developed just after SNHL. 

In the subgroup analysis of glaucoma, both the NTG and ACG revealed a significantly higher incidence in those with SNHL, while the incidence of OAG was only marginally elevated. As the main treatment option for patients with SNHL is corticosteroid administration [36,37], the incidence of OAG (i.e., high-tension glaucoma) should be more prominent due to the effect of steroid on the elevation of IOP, which is known as steroid-induced glaucoma [38]. Nevertheless, patients diagnosed with OAG did not show a significantly elevated numbers in those with SNHL, while NTG, a glaucomatous disorder with normal or even low IOP [39], yielded the highest aHR numerically in SNHL patients. This result further strengthens the concept that higher incidence of glaucoma in patients with SNHL originates from the generalized vulnerable nervous system rather than the effect of steroid-related glaucomatous lesions, since NTG has lower tolerance of mechanical injury [39]. Although ACG also showed an elevated IOP, the glaucomatous damage in ACG often results from the acute pressure effect to the optic nerve head, which needs prompt management [40]. Thus, it is reasonable that a weak nerve in SNHL would be injured by the IOP spike which more commonly contributes to ACG.

Regarding other factors that lead to a higher incidence of glaucoma occurrence, age is a well-known risk factor of glaucoma and the aHR in patients aged 60–79 showed a significantly higher value, which is consistent with findings of previous studies [20]. The insignificant result of patients aged 80 or more could be due to the small group size in the subject population, which might lead to statistical bias. The systemic diseases that correlated with higher glaucoma development including ischemic heart disease and hyperlipidemia, while vascular impairment and ischemia were regarded as risk factors of glaucoma, particularly for the normal-tension subtype [41,42]. Interestingly, the development of glaucoma did not correlate with the longer disease period of SNHL according to the interaction test, implying that the SNHL is not a direct causing factor of glaucoma but an indicator of glaucoma development.

On the epidemiology aspect, about 16 percent of population aged from 20 to 69 years in U.S. suffered from SNHL [10]. Glaucoma revealed has an approximate global prevalence of 3.5 percent [40]. In the current study, the incidence rate of glaucoma in the study group was 43.36 per 100,000 person months, which was significantly higher than the rate of 32.93 per 100,000 person months in the control group. Importantly, the incidence rate of glaucoma in the study group was also numerically higher that the glaucoma incidence rate detected in a previous study using the same research database [43], indicating a prominent incidence of glaucoma in SNHL. As both glaucoma and SNHL influence a large proportion of population, a periodically ophthalmic examination is recommended for patients with SNHL.

There are still certain limitations in the present study. First, the retrospective design of the current study may reduce the homogeneity of the whole study population even after propensity score matching. Moreover, we evaluated claimed data rather than medical records meaning we were missing certain important data like the laterality of disease development and extent of disease severity of both the SNHL and glaucoma, and the results from the pure-tone audiogram, optical coherence tomography and visual field test. Moreover, the use of corticosteroids was not considered in the current study to prevent over-adjustment, which is a prominent risk factor of glaucoma. Still, since the NTG and ACG did not belong to the steroid-induced glaucoma, the significantly higher incidence of these subtypes of glaucoma might imply that steroids play a minor role for glaucoma development in the SNHL population.

## 5. Conclusions

In conclusion, the existence of SNHL is correlated to increased incidence of glaucoma after considering multiple potential risk factors. More specifically, the NTG and ACG are more prominent in those with SNHL compared to the OAG. Accordingly, routine glaucomatous examination should be recommended for those with previous SNHL events. A further large-scale prospective study to evaluate whether the prognosis of glaucoma is affected by the presence of SNHL is required.

## Figures and Tables

**Table 1:** Basic characters of the study and control groups.

Basic Characters	Study	Control	*p* Value
Age			0.2296
<40	1987 (12.67%)	3839 (12.24%)	
40–59	5338 (34.03%)	10,518 (33.53%)	
60–79	6542 (41.71%)	13,362 (42.59%)	
≥80	1819 (11.6%)	3653 (11.64%)	
Sex			0.7436
Male	8480 (54.06%)	17,010 (54.22%)	
Female	7206 (45.94%)	14,362 (45.78%)	
Co-morbidities (before index date)			
Hypertension	7755 (49.44%)	15,826 (50.45%)	0.0394
Diabetes mellitus	3842 (24.49%)	7777 (24.79%)	0.4821
Ischemic heart diseases	3821 (24.36%)	7807 (24.89%)	0.2124
Hyperlipidemia	5849 (37.29%)	11,719 (37.35%)	0.8875
Heart failure	1487 (9.48%)	3109 (9.91%)	0.1383
Cerebrovascular disease	3103 (19.78%)	6199 (19.76%)	0.9543
Dementia	536 (3.42%)	1003 (3.2%)	0.2060
Alzheimer's disease	49 (0.31%)	72 (0.23%)	0.0942
Parkinson's disease	231 (1.47%)	365 (1.16%)	0.0047
Liver disease	5218 (33.27%)	10,513 (33.51%)	0.5947
Rheumatic disease	639 (4.07%)	1237 (3.94%)	0.4945
Kidney disease	6994 (44.59%)	13,973 (44.54%)	0.9216
Hemiplegia or paraplegia	1738 (11.08%)	3489 (11.12%)	0.8927

**Table 2:** Incidence of glaucoma in the study and control groups.

Incidence	Study	Control
Median of follow up months	63	59
Follow up person months	1,024,001	1,964,797
New glaucoma case	444	647
Incidence rate * (95% CI)	43.36 (39.51–47.84)	32.93 (30.49–34.22)
Crude relative risk (95% CI)	1.318 (1.168–1.488)	Reference

* Incidence rate, per 100,000 person months. CI: confidential interval.

**Table 3:** Multiple Cox proportional hazard regression for estimation of adjusted hazard ratios on glaucoma.

Variable	aHR (95% CI)
SNHL (ref: Control)	1.318 (1.168–1.487)
Age (ref: 40–59)	
<40	0.298 (0.197–0.450)
60–79	1.613 (1.387–1.877)
≥80	1.201 (0.949–1.520)
Sex (ref: Female)	
Male	1.044 (0.925–1.178)
Co-morbidities	
Hypertension	1.153 (0.995–1.335)
Diabetes mellitus	1.110 (0.968–1.273)
Ischemic heart diseases	1.225 (1.065–1.409)
Hyperlipidemia	1.346 (1.178–1.537)
Heart failure	0.899 (0.739–1.093)
Cerebrovascular disease	1.004 (0.866–1.164)
Dementia	0.958 (0.676–1.356)
Alzheimer's disease	0.646 (0.158–2.647)
Parkinson's disease	1.202 (0.756–1.913)
Liver disease	1.156 (0.958–1.314)
Rheumatic disease	1.287 (0.990–1.674)
Kidney disease	1.149 (0.867–1.302)
Hemiplegia or paraplegia	0.969 (0.805–1.167)

SNHL: sensorineural hearing loss. aHR: adjusted hazard ratio. CI: confidential interval.

**Table 4:** Multiple Cox proportional hazard regression for estimation of adjusted hazard ratios on different types of glaucoma.

Glaucoma Subtype	aHR (95% CI) for Exposure of SNHL	*p* Value
All glaucoma	1.318 (1.168–1.487)	<0.0001
OAG	1.228 (0.943–1.599)	0.1271
NTG	2.443 (1.561–3.825)	<0.0001
ACG	1.304 (1.053–1.614)	0.0148

aHR: adjusted hazard ratio; CI: confidential interval; SNHL: sensorineural hearing loss; OAG: open angle glaucoma; NTG: normal tension glaucoma; ACG: angle closure glaucoma.

**Table 5:** The sensitivity analysis for the adjusted hazard ratio of glaucoma stratified by follow up time, gender and age subgroups.

Subgroups	Incidence Rate * (95% CI) of Glaucoma		aHR (95% CI)
	Study	Control	
All	43.36 (39.51–47.84)	32.93 (30.49–34.22)	1.318 (1.168–1.487)
Follow up time (Year)			
<2	40.81 (34.52–48.25)	33.74 (29.60–38.46)	1.218 (0.985–1.507)
2–5	47.80 (41.28–55.34)	31.89 (28.02–36.29)	1.498 (1.232–1.822)
≥5	40.67 (34.33–48.17)	33.39 (29.10–38.30)	1.214 (0.976–1.511)
*p* for interaction			0.5372
Gender subgroups			
Male	46.69 (41.32–52.77)	32.45 (29.18–36.09)	1.429 (1.215–1.681)
Female	39.49 (34.21–45.57)	33.48 (29.93–37.44)	1.191 (0.993–1.429)
*p* for interaction			0.1342
Age at index date			
<40	10.44 (6.18–17.62)	4.34 (2.41–7.84)	2.378 (1.066–5.307)
40–59	29.64 (24.54–35.79)	25.05 (21.62–29.02)	1.176 (0.925–1.495)
60–79	62.91 (55.77–70.96)	48.12 (43.61–53.09)	1.306 (1.118–1.526)
≥80	54.69 (42.18–70.9)	34.47 (26.87–44.21)	1.622 (1.131–2.327)
*p* for interaction			0.2663

* Incidence rate, per 100,000 person months; aHR: adjusted hazard ratio; CI: confidential interval.

## References

[1] Frisina R.D., Ding B., Zhu X., Walton J.P. (2016). Age-related hearing loss: Prevention of threshold declines, cell loss and apoptosis in spiral ganglion neurons. Aging.

[2] Chau J.K., Cho J.J., Fritz D.K. (2012). Evidence-based practice: Management of adult sensorineural hearing loss. Otolaryng Clin. N. Am..

[3] Wongrakpanich S., Petchlorlian A., Rosenzweig A. (2016). Sensorineural organs dysfunction and cognitive decline: A review article. Aging Dis..

[4] Paul A., Marlin S., Parodi M., Rouillon I., Guerlain J., Pingault V., Couloigner V., Garabedian E.N., Denoyelle F., Loundon N. (2017). Unilateral sensorineural hearing loss: Medical context and etiology. Audiol Neuro-Otol..

[5] Holy R., Navara M., Dosel P., Fundova P., Prazenica P., Hahn A. (2011). Hyperbaric oxygen therapy in idiopathic sudden sensorineural hearing loss (ISSNHL) in association with combined treatment. Undersea Hyperb. Med..

[6] Di-Stadio A., Dipietro L., Ralli M., Meneghello F., Minni A., Greco A., Stabile M.R., Bernitsas E. (2018). Sudden hearing loss as an early detector of multiple sclerosis: A systematic review. Eur. Rev. Med. Pharmacol. Sci..

[7] Martini A., Castiglione A., Bovo R., Vallesi A., Gabelli C. (2014). Aging, cognitive load, dementia and hearing loss. Audiol. Neuro-Otol..

[8] Scheltens P., Blennow K., Breteler M.M., de Strooper B., Frisoni G.B., Salloway S., Van der Flier W.M. (2016). Alzheimer's disease. Lancet.

[9] Quaranta N., Coppola F., Casulli M., Barulli M.R., Panza F., Tortelli R., Capozzo R., Leo A., Tursi M., Grasso A. (2014). The prevalence of peripheral and central hearing impairment and its relation to cognition in older adults. Audiol. Neuro-Otol..

[10] Golub J.S. (2017). Brain changes associated with age-related hearing loss. Curr. Opin. Otolaryngo..

[11] Jutley G., Luk S.M., Dehabadi M.H., Cordeiro M.F. (2017). Management of glaucoma as a neurodegenerative disease. Neurodegener. Dis..

[12] Huang P., Shi Y., Wang X., Liu M., Zhang C. (2014). Interocular asymmetry of the visual field defects in newly diagnosed normal-tension glaucoma, primary open-angle glaucoma, and chronic angle-closure glaucoma. J. Glaucoma..

[13] Vidal-Sanz M., Salinas-Navarro M., Nadal-Nicolas F.M., Alarcon-Martinez L., Valiente-Soriano F.J., de Imperial J.M., Aviles-Trigueros M., Agudo-Barriuso M., Villegas-Perez M.P. (2012). Understanding glaucomatous damage: Anatomical and functional data from ocular hypertensive rodent retinas. Prog. Retin. Eye. Res..

[14] Almasieh M., Wilson A.M., Morquette B., Cueva Vargas J.L., Di Polo A. (2012). The molecular basis of retinal ganglion cell death in glaucoma. Prog. Retin. Eye. Res..

[15] Liberman M.C., Kujawa S.G. (2017). Cochlear synaptopathy in acquired sensorineural hearing loss: Manifestations and mechanisms. Hearing Res..

[16] Schreiber B.E., Agrup C., Haskard D.O., Luxon L.M. (2010). Sudden sensorineural hearing loss. Lancet.

[17] Kuhn M., Heman-Ackah S.E., Shaikh J.A., Roehm P.C. (2011). Sudden sensorineural hearing loss: A review of diagnosis, treatment, and prognosis. Trends Amplif..

[18] Sara S.A., Teh B.M., Friedland P. (2014). Bilateral sudden sensorineural hearing loss: Review. J. Laryngol. Otol..

[19] Leske M.C., Wu S.Y., Hennis A., Honkanen R., Nemesure B. (2008). Risk factors for incident open-angle glaucoma: The barbados eye studies. Ophthalmology.

[20] Mwanza J.C., Tulenko S.E., Barton K., Herndon L.W., Mathenge E., Hall A., Kim H.Y., Hay-Smith G., Budenz D.L. (2019). Eight-year incidence of open-angle glaucoma in the tema eye survey. Ophthalmology.

[21] Choi J., Kook M.S. (2015). Systemic and ocular hemodynamic risk factors in glaucoma. Biomed. Res. Int..

[22] Lee C.Y., Liu C.H., Chen H.C., Sun C.C., Yao Y.P., Chao S.C. (2019). Correlation between basal macular circulation and following glaucomatous damage in progressed high-tension and normal-tension glaucoma. Ophthalmic Res..

[23] Kujawa S.G., Liberman M.C. (2015). Synaptopathy in the noise-exposed and aging cochlea: Primary neural degeneration in acquired sensorineural hearing loss. Hearing Res..

[24] Vaden K.I., Matthews L.J., Dubno J.R. (2018). Transient-evoked otoacoustic emissions reflect audiometric patterns of age-related hearing loss. Trends Hear..

[25] Ungar O.J., Handzel O., Santos F. (2018). Rate of spiral ganglion cell loss in idiopathic sudden sensorineural hearing loss. Hear. Res..

[26] Tavanai E., Mohammadkhani G. (2017). Role of antioxidants in prevention of age-related hearing loss: A review of literature. Eur. Arch. Oto-Rh..

[27] Ding D., Jiang H., Chen G.D., Longo-Guess C., Muthaiah V.P., Tian C., Sheppard A., Salvi R., Johnson K.R. (2016). N-acetyl-cysteine prevents age-related hearing loss and the progressive loss of inner hair cells in gamma-glutamyl transferase 1 deficient mice. Aging.

[28] Alqawlaq S., Flanagan J.G., Sivak J.M. (2019). All roads lead to glaucoma: Induced retinal injury cascades contribute to a common neurodegenerative outcome. Exp. Eye. Res..

[29] Mancino R., Martucci A., Cesareo M., Giannini C., Corasaniti M.T., Bagetta G., Nucci C. (2018). Glaucoma and alzheimer disease: One age-related neurodegenerative disease of the brain. Curr. Neuropharmacol..

[30] Lee C.S., Larson E.B., Gibbons L.E., Lee A.Y., McCurry S.M., Bowen J.D., McCormick W.C., Crane P.K. (2019). Associations between recent and established ophthalmic conditions and risk of alzheimer's disease. Alzheimers. Dement..

[31] Lopez-Escamez J.A., Carey J., Chung W.H., Goebel J.A., Magnusson M., Mandala M., Newman-Toker D.E., Strupp M., Suzuki M., Trabalzini F. (2015). Diagnostic criteria for meniere's disease. J. Vestibul. Res-Equil..

[32] Pyykko I., Nakashima T., Yoshida T., Zou J., Naganawa S. (2013). Meniere's disease: A reappraisal supported by a variable latency of symptoms and the mri visualisation of endolymphatic hydrops. BMJ Open.

[33] Nakashima T., Sone M., Teranishi M., Yoshida T., Terasaki H., Kondo M., Yasuma T., Wakabayashi T., Nagatani T., Naganawa S. (2012). A perspective from magnetic resonance imaging findings of the inner ear: Relationships among cerebrospinal, ocular and inner ear fluids. Auris Nasus Larynx.

[34] Bozkurt M.K., Ozturk B.T., Kerimoglu H., Ersan I., Arbag H., Bozkurt B. (2011). Association of age-related macular degeneration with age-related hearing loss. J. Laryngol. Otol..

[35] Hogewind B.F., Pennings R.J., Hol F.A., Kunst H.P., Hoefsloot E.H., Cruysberg J.R., Cremers C.W. (2010). Autosomal dominant optic neuropathy and sensorineual hearing loss associated with a novel mutation of wfs1. Mol. Vis..

[36] Metrailer A.M., Babu S.C. (2016). Management of sudden sensorineural hearing loss. Curr. Opin. Otolaryngo..

[37] Stachler R.J., Chandrasekhar S.S., Archer S.M., Rosenfeld R.M., Schwartz S.R., Barrs D.M., Brown S.R., Fife T.D., Ford P., Ganiats T.G. (2012). Clinical practice guideline: Sudden hearing loss. Otolaryng. Head. Neck..

[38] Fini M.E., Schwartz S.G., Gao X., Jeong S., Patel N., Itakura T., Price M.O., Price F.W., Varma R., Stamer W.D. (2017). Steroid-induced ocular hypertension/glaucoma: Focus on pharmacogenomics and implications for precision medicine. Prog. Retin. Eye. Res..

[39] Killer H.E., Pircher A. (2018). Normal tension glaucoma: Review of current understanding and mechanisms of the pathogenesis. Eye (Lond)..

[40] Jonas J.B., Aung T., Bourne R.R., Bron A.M., Ritch R., Panda-Jonas S. (2017). Glaucoma. Lancet.

[41] Mallick J., Devi L., Malik P.K., Mallick J. (2016). Update on normal tension glaucoma. J. Ophthalmic Vis. Res..

[42] Evangelho K., Mogilevskaya M., Losada-Barragan M., Vargas-Sanchez J.K. (2019). Pathophysiology of primary open-angle glaucoma from a neuroinflammatory and neurotoxicity perspective: A review of the literature. Int. Ophthalmol..

[43] Huang J.Y., Su C.C., Wang T.H., Tsai I.J. (2019). Migraine and increased risk of developing open angle glaucoma: A population-based cohort study. BMC Ophthalmol..

